# MERS-CoV‒Specific T-Cell Responses in Camels after Single MVA-MERS-S Vaccination

**DOI:** 10.3201/eid2906.230128

**Published:** 2023-06

**Authors:** Christian Meyer zu Natrup, Lisa-Marie Schünemann, Giulietta Saletti, Sabrina Clever, Vanessa Herder, Sunitha Joseph, Marina Rodriguez, Ulrich Wernery, Gerd Sutter, Asisa Volz

**Affiliations:** University of Veterinary Medicine Hannover, Hanover, Germany (C. Meyer zu Natrup, L.-M. Schünemann, G. Saletti, S. Clever, A. Volz);; University of Glasgow, Glasgow, Scotland, UK (V. Herder);; Central Veterinary Research Laboratory, Dubai, United Arab Emirates (S. Joseph, M. Rodriguez, U. Wernery);; Ludwig Maximilian University Munich, Munich, Germany (G. Sutter)

**Keywords:** MERS-CoV, Middle East respiratory syndrome coronavirus, coronavirus, viruses, dromedary camels, T-cell responses, ELISPOT assay, MVA, modified vaccinia virus Ankara, MVA-MERS-S, MVA expressing full-length MERS-CoV spike protein as antigen, vaccination, respiratory infections, spike protein, zoonoses, Dubai

## Abstract

We developed an ELISPOT assay for evaluating Middle East respiratory syndrome coronavirus (MERS-CoV)‒specific T-cell responses in dromedary camels. After single modified vaccinia virus Ankara-MERS-S vaccination, seropositive camels showed increased levels of MERS-CoV‒specific T cells and antibodies, indicating suitability of camel vaccinations in disease-endemic areas as a promising approach to control infection.

The Middle East respiratory syndrome coronavirus (MERS-CoV) is a betacoronavirus that is of special interest for public health. Dromedary camels have been identified as natural animal reservoirs, with >90% MERS-CoV seroprevalence reported in Middle East countries (*1*–*4*). Such permanent viral circulation within camel herds poses a constant threat of zoonotic transmission into human populations (*5*). Thus, a potentially useful approach to prevent MERS-CoV zoonoses focuses on vaccination-based reduction of spill over events from camels as a classical One Health approach (*6*,*7*).

Besides antibody responses, MERS-CoV–specific T cells probably play a major role in rapid viral clearance and long-lasting immunity against MERS-CoV infections (*8*). Although serologic assays were rapidly developed, established T-cell assays for camels are still lacking, yet urgently needed for contact tracing, epidemiology, and vaccine evaluation studies. Several MERS-CoV‒specific vaccine candidates are under investigation and use different platforms, such as DNA vaccines or adenoviral vectors (*9*–*12*). A promising experimental vaccine for use in camels is recombinant modified vaccinia virus Ankara (MVA) expressing full-length MERS-CoV spike protein as antigen (MVA-MERS-S) (*13*). Experimental vaccination with MVA-MERS-S in dromedaries can induce protective immunity to MERS-CoV (*14*). Moreover, MVA-MERS-S proved safe and immunogenic in a phase Ia/b clinical study in humans (*15*). The aim of this exploratory study in Dubai, United Arab Emirates, where enzootic MERS-CoV is prevalent, was to establish an assay for detecting MERS-CoV‒specific T cells in dromedary camels under field conditions.

## The Study

To investigate the effect of MVA-MERS-S vaccination in naive or previously infected animals, we divided 12 adult dromedary camels into 2 cohorts: naive and MERS-CoV seropositive solely based on presence of MERS-S IgG (by ELISA) before vaccination. Eight camels had antibody titers relevant for seroconversion (optical density [OD] ratio >1.1), indicating previous MERS-CoV infection, whereas the remaining 4 camels had no MERS-specific antibodies (Table 1).

**Table 1:** MERS-CoV seroprevalence in 12 dromedary camels before vaccination, Dubai, United Arab Emirates*

Camel ID	Optical density ratio (ELISA)	MERS-CoV infection status/cohort
1	0.07	Naive
2	2.09	Seropositive
3	2.97	Seropositive
4	4.00	Seropositive
5	0.06	Naive
6	0.07	Naive
7	0.05	Naive
8	4.11	Seropositive
9	3.42	Seropositive
10	4.59	Seropositive
11	2.48	Seropositive
12	3.22	Seropositive

*ID, identification; MERS-CoV, Middle East respiratory syndrome coronavirus.

**Figure 1:**
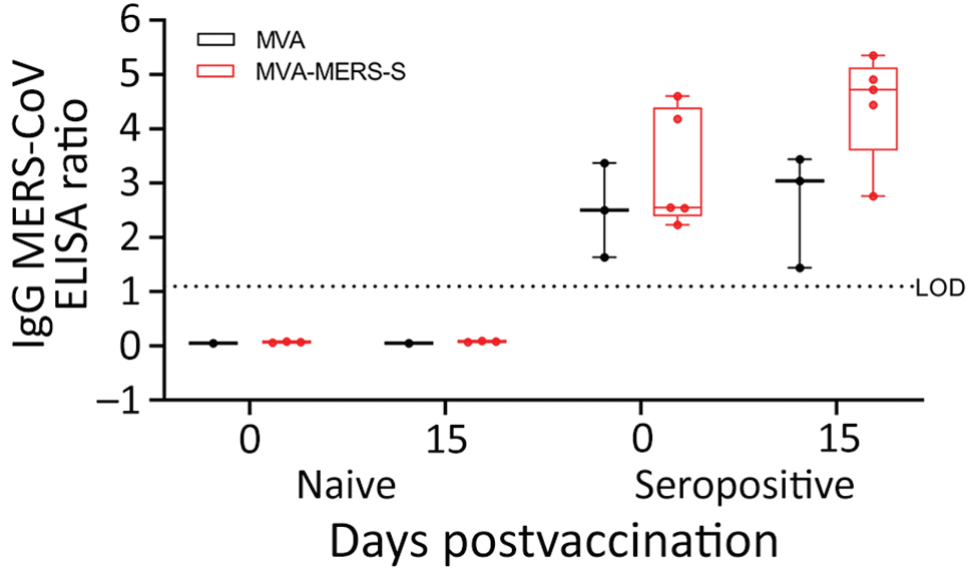
Antigen-specific humoral immunity after MVA-MERS-S vaccination in dromedary camels, Dubai, United Arab Emirates. MERS-CoV seropositive and naive dromedary camels were immunized once with 2.5 x 10^8^ plaque-forming units of MVA-MERS-S or MVA as a vector control. Serum samples were collected on day 0 and on day 15 after single-shot vaccination. Black indicates serum samples analyzed for MERS-CoV S1 IgG by ELISA of MVA–vaccinated animals and red indicates for MVA-MERS-S–vaccinated animals. Box plots show individual values (dots), median values (horizontal lines within boxes), first and third quartiles (box tops and bottoms), and minimums and maximums of value distribution (error bars). LOD, limit of detection; MERS-CoV, Middle East respiratory syndrome coronavirus; MVA, modified vaccinia virus Ankara; MVA-MERS-S, modified vaccinia virus Ankara expressing full-length MERS-CoV spike protein as antigen.

Camels were either vaccinated with MVA-MERS-S or MVA as a control by using intramuscular inoculation (dose 2.5 × 10^8^ PFU/2 mL) (Table 2). Animals and application sites were monitored and scored daily for an observation period of 22 days. No clinical signs or potential side effects were observed (data not shown). Analysis of the IgG response at the day of vaccination and 15 days later (Figure 1) showed no differences in MERS-CoV–specific antibodies in naive camels (MVA– and MVA-MERS-S–vaccinated camels).

**Table 2:** Cohorts of dromedary camels by MERS-CoV seroprevalence and vaccine candidate used Dubai, United Arab Emirates*

Category	Type	Vaccine candidate		Total
		MVA	MVA-MERS-S	
MERS-CoV infection	Naive	1	3	4
	Seropositive	3	5	8
	Total	4	8	12

*MERS-CoV, Middle East respiratory syndrome coronavirus; MVA, modified vaccinia virus Ankara; MVA-MERS-S, modified vaccinia virus Ankara expressing full-length MERS-CoV spike protein as antigen.

One seropositive animal vaccinated with MVA showed an increased optical density (OD) ratio of 0.54, whereas the other 2 animals showed no difference or a decreased ratio of 0.19. Seropositive camels vaccinated with MVA-MERS-S (n = 5) mounted increased levels of MERS binding antibodies, with a mean titer (OD ratio) of 4.44 on day 15 compared with 3.22 at day 0 postvaccination. Two MVA-MERS-S vaccinated camels from seropositive animals showed an increased OD ratio >2.4.

To assess T-cell responses, we prepared peripheral blood mononuclear cells (PBMCs) from blood plus EDTA on different days postvaccination during the observation period. PBMCs were restimulated with 2 pools of overlapping peptides comprising either the S1 or S2 subunit of MERS-CoV spike glycoprotein (Appendix Figure) analyzed by using interferon (IFN) γ ELISpot assays.

**Figure 2:**
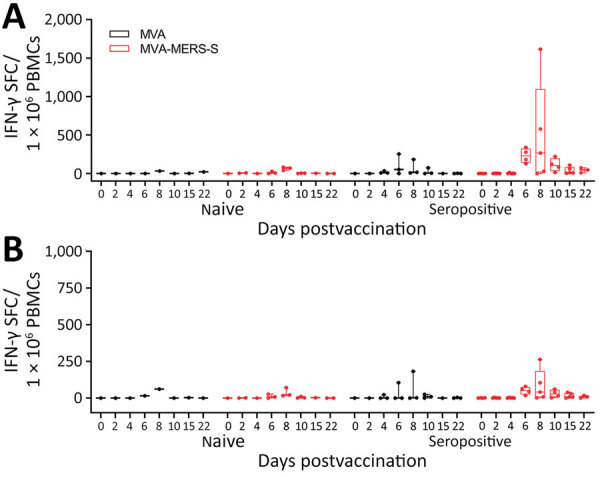
Antigen-specific cellular immunity after MVA-MERS-S vaccination in dromedary camels, Dubai, United Arab Emirates. PBMCs were isolated from blood samples on different days post‒single-shot vaccination and IFN-γ SFCs were measured by ELISpot assay after restimulation of PBMCs from different time points with overlapping peptides comprising the MERS-CoV S1 (A) and MERS-CoV-S2 (B) protein subunit. Box plots show individual values (dots), median values (horizontal lines within boxes), first and third quartiles (box tops and bottoms), and minimums and maximums of value distribution (error bars). IFN-γ, interferon-γ; MERS-CoV, Middle East respiratory syndrome coronavirus; MVA, modified vaccinia virus Ankara; MVA-MERS-S, modified vaccinia virus Ankara expressing full-length MERS-CoV spike protein as antigen; PBMCs, peripheral blood mononuclear cells; SFCs, spot-forming T cells.

After S1 peptide pool stimulation, we detected no IFN-γ‒producing cells in the MVA-vaccinated naive animals (Figure 2, panel A). MVA-MERS-S vaccinated naive animals (n = 3) showed detectable levels of S1-specific T cells on day 6 postvaccination (mean 11.1 spot-forming T cells [SFC]/10^6^ PBMCs), which further increased until day 8 postvaccination (mean 63.3 IFN-γ SFC/10^6^ PBMCs).

MVA-vaccinated seropositive animals showed negligible levels of IFN-γ‒producing cells, except for 1 animal that had S1-specific T cells on days 6 and 8 postvaccination (mean 217.8 IFN-γ SFC/10^6^ PBMCs). Seropositive MVA-MERS-S–vaccinated animals had substantially higher activated S1-specific T-cell levels starting on day 6 postvaccination (mean 230.6 IFN-γ SFC/10^6^ PBMCs), further increasing on day 8 postvaccination (mean 497.8 IFN-γ SFC/10^6^ PBMCs). Subsequently, S1-specific T-cell levels decreased on day 10 (mean 110 IFN-γ SFC/10^6^ PBMCs), until reaching relatively low levels at day 22 postvaccination (mean 42.8 IFN-γ SFC/10^6^ PBMCs).

Upon S2 peptide stimulation, we detected lower levels of IFN-γ‒producing cells compared with S1 peptide stimulation (Figure 2, panel B). The MVA-vaccinated naive camels had low levels of IFN-γ‒producing cells on day 6 and 8 postvaccination (mean 38.3 IFN-γ SFC/10^6^ PBMCs). MVA-MERS-S-vaccinated naive animals showed low responses in all animals; mean levels of 11.9 IFN-γ SFC/10^6^ PBMCs on day 6 postvaccination increased to 36.3 IFN-γ SFC/10^6^ PBMCs on day 8 postvaccination, then decreased again by day 22 postvaccination.

Two MVA-vaccinated seropositive animals mounted no detectable levels of S2-specific T cells. The same seropositive animal mounting S1-specific T cells revealed increased levels of S2-specific T cell activation on day 6 and 8 postvaccination (mean 143.6 IFN-γ SFC/10^6^ PBMCs). All seropositive MVA-MERS-S–vaccinated animals had levels of S2-specific T cells that increased on day 6 postvaccination (mean 50.6 IFN-γ SFC/10^6^ PBMCs), further increasing on day 8 postvaccination (mean 84 IFN-γ SFC/10^6^ PBMCs). Again, the S2-specific T cells subsequently decreased by day 22 postvaccination (mean 8.9 IFN-γ SFC/10^6^ PBMCs).

## Conclusions

This exploratory study confirms the presence of MERS-S–specific T cells in dromedary camels after a single MVA-MERS-S vaccination under field conditions as analyzed by IFN-γ ELISPOT assay. Previous infection seems not to hamper the practicability or value of vaccination trials because specific T cells were immunologically boosted in seropositive camels. These data are consistent with a recent study of humoral boost effects in seropositive camels after vaccination with a chimpanzee adenoviral vector-based MERS-CoV vaccine (*12*). This finding is relevant because serum antibodies are considered to reduce viral replication (*6*). MVA-MERS-S vaccination also reactivated humoral immune responses in seropositive camels. Our previous study confirmed that MERS-CoV-S–specific antibodies correlate with reduced viral excretion in camels (*14*). These preliminary results could have major implications for implementing future MVA-MERS-S camel vaccination studies in disease-endemic areas.

Naive MVA-MERS-S‒vaccinated animals mounted fewer MERS-CoV-S–specific T cells than seropositive animals and failed to show S-specific antibodies after single MVA-MERS-S vaccination. Thus, further optimizing MVA-MERS-S‒induced immunogenicity would require modifying vaccination strategies under field conditions, such as prime-boost vaccination regimens or alternative applications including intranasal immunization.

Although it is unlikely for the specific T cells detected in 1 seropositive and 1 naive camel after MVA vaccination, we cannot rule out a field infection between vaccination and sample preparation. Rather, we hypothesize that the seropositive animal could have remounted a cellular immune response caused by MVA-induced immune activation and potential coactivation of S-peptide specific T cells from previous MERS-CoV infection. In the naive camel, which did not seroconvert or mount S1-specific responses, nonspecific reactions could explain the detection of IFN-γ SFC.

The first limitation for this proof-of-concept study is that it was conducted as an exploratory study to evaluate MERS-CoV‒specific T cells in a few camels and provide a basis for further evaluation of camel vaccination in disease-endemic areas. To verify the potential protective capacity of vaccine-induced immune responses under field conditions, it will be essential to also characterize the infection status and demonstrate reduced virus excretion in vaccinated, subsequently infected animals. Future field studies could be based on MVA-MERS-S vaccination, not only in prime-only immunization cohorts but also in prime-boost applications, especially in juvenile animals, the probable main drivers of MERS-CoV transmission in camel populations (*6*). Our findings should contribute to establishing an advanced method for evaluating MERS-CoV‒specific cellular immunity in dromedary camels.

Appendix Additional information on MERS-CoV‒specific T-cell responses in camels after single MVA-MERS-S vaccination.

## References

[1] Reusken CB, Haagmans BL, Müller MA, Gutierrez C, Godeke GJ, Meyer B, et al. Middle East respiratory syndrome coronavirus neutralising serum antibodies in dromedary camels: a comparative serological study. Lancet Infect Dis. 2013;13:859–66. 10.1016/S1473-3099(13)70164-6 23933067 PMC7106530

[2] Meyer B, Müller MA, Corman VM, Reusken CB, Ritz D, Godeke GJ, et al. Antibodies against MERS coronavirus in dromedary camels, United Arab Emirates, 2003 and 2013. Emerg Infect Dis. 2014;20:552–9. 10.3201/eid2004.131746 24655412 PMC3966379

[3] Haagmans BL, Al Dhahiry SH, Reusken CB, Raj VS, Galiano M, Myers R, et al. Middle East respiratory syndrome coronavirus in dromedary camels: an outbreak investigation. Lancet Infect Dis. 2014;14:140–5. 10.1016/S1473-3099(13)70690-X 24355866 PMC7106553

[4] Müller MA, Meyer B, Corman VM, Al-Masri M, Turkestani A, Ritz D, et al. Presence of Middle East respiratory syndrome coronavirus antibodies in Saudi Arabia: a nationwide, cross-sectional, serological study. Lancet Infect Dis. 2015;15:559–64. 10.1016/S1473-3099(15)70090-3 25863564 PMC7185864

[5] Mohd HA, Al-Tawfiq JA, Memish ZA. Middle East respiratory syndrome coronavirus (MERS-CoV) origin and animal reservoir. Virol J. 2016;13:87. 10.1186/s12985-016-0544-0 27255185 PMC4891877

[6] Meyer B, Juhasz J, Barua R, Das Gupta A, Hakimuddin F, Corman VM, et al. Time course of MERS-CoV infection and immunity in dromedary camels. Emerg Infect Dis. 2016;22:2171–3. 10.3201/eid2212.160382 27224315 PMC5189137

[7] Hemida MG, Chu DK, Poon LL, Perera RA, Alhammadi MA, Ng HY, et al. MERS coronavirus in dromedary camel herd, Saudi Arabia. Emerg Infect Dis. 2014;20:1231–4. 10.3201/eid2007.140571 24964193 PMC4073860

[8] Zhao J, Alshukairi AN, Baharoon SA, Ahmed WA, Bokhari AA, Nehdi AM, et al. Recovery from the Middle East respiratory syndrome is associated with antibody and T-cell responses. Sci Immunol. 2017;2:eaan5393. 10.1126/sciimmunol.aan5393 28778905 PMC5576145

[9] Muthumani K, Falzarano D, Reuschel EL, Tingey C, Flingai S, Villarreal DO, et al. A synthetic consensus anti-spike protein DNA vaccine induces protective immunity against Middle East respiratory syndrome coronavirus in nonhuman primates. Sci Transl Med. 2015;7:301ra132. 10.1126/scitranslmed.aac7462 26290414 PMC4573558

[10] Modjarrad K, Roberts CC, Mills KT, Castellano AR, Paolino K, Muthumani K, et al. Safety and immunogenicity of an anti-Middle East respiratory syndrome coronavirus DNA vaccine: a phase 1, open-label, single-arm, dose-escalation trial. Lancet Infect Dis. 2019;19:1013–22. 10.1016/S1473-3099(19)30266-X 31351922 PMC7185789

[11] Munster VJ, Wells D, Lambe T, Wright D, Fischer RJ, Bushmaker T, et al. Protective efficacy of a novel simian adenovirus vaccine against lethal MERS-CoV challenge in a transgenic human DPP4 mouse model. NPJ Vaccines. 2017;2:28. 10.1038/s41541-017-0029-1 29263883 PMC5643297

[12] Alharbi NK, Qasim I, Almasoud A, Aljami HA, Alenazi MW, Alhafufi A, et al. Humoral immunogenicity and efficacy of a single dose of ChAdOx1 MERS vaccine candidate in dromedary camels. Sci Rep. 2019;9:16292. 10.1038/s41598-019-52730-4 31705137 PMC6841732

[13] Volz A, Kupke A, Song F, Jany S, Fux R, Shams-Eldin H, et al. Protective efficacy of recombinant modified vaccinia virus Ankara delivering Middle East respiratory syndrome coronavirus spike glycoprotein. J Virol. 2015;89:8651–6. 10.1128/JVI.00614-15 26018172 PMC4524222

[14] Haagmans BL, van den Brand JM, Raj VS, Volz A, Wohlsein P, Smits SL, et al. An orthopoxvirus-based vaccine reduces virus excretion after MERS-CoV infection in dromedary camels. Science. 2016;351:77–81. 10.1126/science.aad1283 26678878

[15] Koch T, Dahlke C, Fathi A, Kupke A, Krähling V, Okba NMA, et al. Safety and immunogenicity of a modified vaccinia virus Ankara vector vaccine candidate for Middle East respiratory syndrome: an open-label, phase 1 trial. Lancet Infect Dis. 2020;20:827–38. 10.1016/S1473-3099(20)30248-6 32325037 PMC7172913

