# Supplementary material for: MERS-CoV‒Specific T-Cell Responses in Camels after Single MVA-MERS-S Vaccination
Source: Emerg Infect Dis. 2023 Jun;29(6):1236–9. doi: 10.3201/eid2906.230128 (PMC10202854; doi:10.3201/eid2906.230128)
Supplement: Appendix — Additional information on MERS-CoV‒specific T-cell responses in camels after single MVA-MERS-S vaccination. [file 23-0128-Techapp-s1.pdf]

Article DOI: https://doi.org/10.3201/eid2906.220138

EID cannot ensure accessibility for supplementary materials supplied by authors. Readers who have difficulty accessing supplementary content should contact the authors for assistance.

MERS-CoV–Specific T-Cell Responses in Camels after Single MVA-MERS-S Vaccination

Appendix

Materials and Methods

Vaccine Candidate

The vaccine candidate used in this proof-of-concept study is MVA-MERS-S, a recombinant Modified Vaccinia Virus Ankara, as a safe vaccine platform expressing the full-length MERS-CoV spike (S) protein. As described by Song et al. (*1*), the vaccine candidate was constructed as follows: cDNA with the whole gene sequence of MERS-CoV-S was provided by performing DNA synthesis (Invitrogen Life Technology, Regensburg, Germany) and modified by inserting silent mutations for vaccinia virus transcription. The MERS-CoV-S coding sequences were integrated into a MVA vector plasmid under the transcriptional control of the vaccinia virus early/late promoter PmH5. For selection of the correctly constructed recombinant MVA-MERS-S, the fluorescent marker gene mCherry was also placed in the MVA vector plasmid (under transcriptional control of the vaccinia virus late promoter P11). The MVA vector plasmid was further inserted into the deletion site Del III in the MVA genome by homologous recombination. The fluorescent marker protein mCherry introduced while cloning enabled d isolation of recombinant MVA-MERS-S by using fluorescence. The mCherry marker was removed by marker gene deletion using repetitive sequences. To further characterize the vaccine candidate, PCR analysis and multiple-step growth analysis were performed, confirming the genetic integrity, stability and safety of MVA-MERS-S.

MVA-Based Vaccination in Dromedary Camels in Dubai

Adult dromedary camels (n = 12) from the Central Veterinary Research Laboratory in Dubai, UAE were housed in different enclosures and had unlimited access to water and food. All of the animals underwent a comprehensive general examination and showed healthy general clinical conditions. The animals were immunized with an intramuscular single-shot-vaccination of 2.5×10^8 PFU/2 mL. Eight of the twelve animals were vaccinated with the recombinant MVA-MERS-S and the other four animals with the non-recombinant MVA as a viral vector control.

IgG ELISA

Serum samples from the selected dromedary camels were collected on the day of vaccination and 15 days post vaccination. MERS-CoV seroprevalence was analyzed by using the Anti-MERS-CoV-ELISA Camel (IgG) kit from EUROIMMUN. The commercial ELISA kit is based on the MERS-CoV S1 subunit as described by Drosten et al. (*2*). Diluted serum samples (1:101 in sample buffer) were first incubated at 37°C for 30 minutes in microplate wells coated with recombinant structural MERS-CoV S1 protein. For detection, an enzyme-labeled anti-camel IgG was then added to the wells in a second incubation step (37°C, 30 minutes). Color-coded substrate was finally added to the wells for 15 minutes at room temperature leading to a color reaction. In between, wash steps were included. The extinction value was measured at OD 450 nm light and semiquantitative evaluations were performed by using the ratio values (sample value extinction over calibrator value extinction). As recommended by EUROIMMUN, a ratio <0.8 was set as a negative result and a ratio ≥ 1.1 as positive.

Enzyme-Linked Immunospot (ELISpot)

Peripheral blood mononuclear cells (PBMCs) were isolated from EDTA blood directly after sampling by density gradient centrifugation on day 0, 2, 4, 6, 8, 10, 15 and 22 post vaccination and stored frozen. Later on, samples from different timepoints from individual camels were quickly thawed at 37°C, washed, and resuspended in RPMI 1640 medium (SIGMA-ALDRICH, Taufkirchen, Germany) supplemented with 10% heat inactivated FCS, 1% Penicillin-Streptomycin, 1% non-essential amino acids and 1% vitamins. The cells were counted with the TC20 Automated Cell Counter (Bio-Rad Laboratories, Feldkirchen, Germany) and 3×10^5 live PBMCs per well were seeded into 96-well round bottom plates (Sarstedt, Nümbrecht, Germany) before adding the stimulants. For PBMC stimulation, peptides that can directly bind to MHC I and MHC II molecules on the APC surface were used as an established method for stimulating

both CD8 and CD4 T cells. We used two different peptide pools from the S1 and S2 domains comprising 168 overlapping peptides each (1µg peptide/ml RPMI 1640) providing the whole spike glycoprotein of MERS-CoV (JPT Peptide Technologies, Berlin, Germany). Each peptide consists of 15 aa (15-mers) overlapping in 11 aa with the following peptide (Supplemental Figure 1). PBMCs stimulated with phorbol myristate acetate and ionomycin (SIGMA-ALDRICH, Taufkirchen, Germany) and non-stimulated cells were used as positive and negative controls. The cells were then transferred onto PVDF membrane plates previously coated with mouse anti-bovine IFN-γ monoclonal antibody (bIFNγ-I; Mabtech, Nacka, Sweden) and incubated for 24 hours at 37°C. After removing the inoculate, biotinylated mouse anti-bovine IFN-γ monoclonal antibody (PAN), streptavidin-ALP and BCIP/NBT-plus substrate were added onto the ELISpot plates in this order with washing steps in between. For scanning and counting the spots we used the automated ELISpot Reader ImmunoSpot S6 ULTIMATE UV Image Analyzer (Immunospot, Bonn, Germany) and ImmunoSpot 7.0.20.1 as the reader software version.

References

1. Song F, Fux R, Provacia LB, Volz A, Eickmann M, Becker S, et al. Middle East respiratory syndrome coronavirus spike protein delivered by modified vaccinia virus Ankara efficiently induces virus-neutralizing antibodies. J Virol. 2013;87:11950–4. PubMed https://doi.org/10.1128/JVI.01672-13

2. Drosten C, Meyer B, Müller MA, Corman VM, Al-Masri M, Hossain R, et al. Transmission of MERS-coronavirus in household contacts. N Engl J Med. 2014;371:828–35. PubMed https://doi.org/10.1056/NEJMoa1405858

MIHSVFLLMF	LLTPTESYVD	VGPDSVKSAC	IEVDIQQTFF	DKTWPRPIDV	SKADGIIYPQ
GRTYSNITIT	YQGLFPYQGD	HGDMYVYSAG	HATGTTPQKL	FVANYSQDVK	QFANGFVVRI
GAAANSTGTV	IISPSTSATI	RKIYPAFMLG	SSVGNFSDGK	MGRFFNHTLV	LLPDGCGTLL
RAFYCILEPR	SGNHCPAGNS	YTSFATYHTP	ATDCSDGNYN	RNASLNSFKE	YFNLRNCTFM
YTYNITEDEI	LEWFGITQTA	QGVHLFSSRY	VDLYGGNMFQ	FATLPVYDTI	KYYSIIPHSI
RSIQSDRKAW	AAFYVYKLQP	LTFLLDFSVD	GYIRRAIDCG	FNDLSQLHCS	YESFDVESGV
YSVSSFEAKP	SGSVVEQAEG	VECDFSPLLS	GTPPQVYNFK	RLVFTNCNYN	LTKLLSLFSV
NDFTCSQISP	AAIASNCYSS	LILDYFSYPL	SMKSDLSVSS	AGPISQFNYK	QSFSNPTCLI
LATVPHNLTT	ITKPLKYSYI	NKCSRFLSDD	RTEVPQLVNA	NQYSPCVSIV	PSTVWEDGDY
YRKQLSPLEG	GGWLVASGST	VAMTEQLQMG	FGITVQYGTD	TNSVCPKLEF	ANDTKIASQL
GNCVEYSLYG	VSGRGVFQNC	TAVGVRQQRF	VYDAYQNLVG	YYSDDGNYYC	LRACVSVPVS
VIYDKETKTH	ATLFGSVACE	HISSTMSQYS	RSTRSMLKRR	DSTYGPLQTP	VGCVLGLVNS
SLFVEDCKLP	LGQSLCALPD	TPSTLTPRSV	RSVPGEMRLA	SIAFNHPIQV	DQLNSSYFKL
SIPTNFSFGV	TQEYIQTTIQ	KVTVDCKQYV	CNGFQKCEQL	LREYGQFCSK	INQALHGANL
RQDDSVRNLF	ASVKSSQSSP	IIPGFGGDFN	LTLLEPVSIS	TGSRSARSAI	EDLLFDKVTI
ADPGYMQGYD	DCMQQGPASA	RDLICAQYVA	GYKVLPPLMD	VNMEAAYTSS	LLGSIAGVGW
TAGLSSFAAI	PFAQSIFYRL	NGVGITQQVL	SENQKLIANK	FNQALGAMQT	GFTTTNEAFH
KVQDAVNNNA	QALSKLASEL	SNTFGAISAS	IGDIIQRLDV	LEQDAQIDRL	INGRLTTLNA
FVAQQLVRSE	SAALSAQLAK	DKVNECVKAQ	SKRSGFCGQG	THIVSFVVNA	PNGLYFMHVG
YYPSNHIEVV	SAYGLCDAAN	PTNCIAPVNG	YFIKTNNTRI	VDEWSYTGSS	FYAPEPITSL
NTKYVAPQVT	YQNISTNLPP	PLLGNSTGID	FQDELDEFFK	NVSTSIPNFG	SLTQINTTLL
DLTYEMLSLQ	QVVKALNESY	IDLKELGNYT	YYNKWPWYIW	LGFIAGLVAL	ALCVFFILCC
TGCGTNCMGK	LKCNRCCDRY	EEYDLEPHKV	HVH		

Appendix Figure. Protein sequence of MERS-CoV spike (S) protein used for PBMC stimulation. The MERS-CoV spike glycoprotein comprises 1353 aa (aa) with two domains, S1 (underlined in yellow) and S2 (underlined in blue). For camel PBMC stimulation, two peptide pools (S1 and S2), consisting of 168 overlapping peptides each, were derived from the MERS-CoV S protein sequence. Each single peptide consists of 15 aa (15-mers) overlapping in 11 aa with the following peptide.
